# EU’s Extraterritorial Obligations for Global Medicine Access Under the Convention on the Rights of Persons with Disability (CPRD)

**DOI:** 10.1017/jme.2025.10144

**Published:** 2025

**Authors:** Katrina Perehudoff

**Affiliations:** 1 University of Amsterdam Amsterdam Law School, Law for Health and Life, Netherlands; 2 Amsterdam Institute for Global Health and Development, Netherlands; 3 Amsterdam Centre for European Studies, University of Amsterdam; 4 Amsterdam Centre for European Law and Governance, University of Amsterdam; 5 Medicines Law & Policy, Amsterdam, the Netherlands

**Keywords:** access to medicines, disability rights, pharmaceuticals, European Union, intellectual property

## Abstract

Equitable access to medicines is vital for people with disabilities to receive effective, affordable, and quality treatment, helping preserve functionality, prevent further disability, and promote social and economic inclusion. This paper explores the specific medicine needs of people with disabilities in low- and middle-income countries (LMICs), focusing on the European Union’s (EU) extraterritorial legal obligations under the Convention on the Rights of Persons with Disabilities (CRPD). As the first regional international organization to accede to a UN human rights treaty, the EU offers a unique case for examining how international legal commitments extend beyond its borders. The paper outlines a legal framework based on the CRPD to assess the EU’s responsibilities for ensuring access to medicines globally. This framework is applied to two case studies: the EU’s internal joint COVID-19 vaccine procurement strategy and its external BioNTainer initiative for vaccine production in Africa under Team Europe. The analysis finds that the EU falls short of its CRPD obligations, particularly in areas of technology transfer and intellectual property sharing, which are essential for equitable global vaccine access. The paper concludes that the EU’s current actions do not fulfill its human rights commitments to people with disabilities in LMICs.

## 1. Introduction

In the last decade, several breakthrough medicines have entered the global market, transforming the potential for people with disabilities to manage and treat conditions that, if left suboptimal or untreated, would have caused them further disability, loss of independence, and reduced quality of life. Consider, for example, medicines such as Orkambi (lumacaftor-ivacaftor), Spinraza (nusinersen), and Zolgensma (onasemnogene abeparvovec) for genetic conditions such as cystic fibrosis and spinal muscular atrophy. These medicines slow “irreversible organ damage” and hold promise for delaying debilitating disease progression and death if administered at a young age and if the body responds to treatment.^1^ Similarly, infectious disease medicines such as Sirturo (bedaquiline) and Sovaldi (sofosbuvir) for multi-drug-resistant tuberculosis and hepatitis C, respectively, have improved quality of life and other patient-reported outcomes when compared to the “grueling” alternative therapies with lower success rates.^2^ Besides their shared potential to revolutionize therapy for these patients and prevent further disability, these medicines also all have an exorbitant price tag in most countries. Prices of these medicines were so sizable that health systems responded by rationing access or not covering the medicine altogether, leaving patients to choose to pay out-of-pocket for the product.^3^ Advocates launched campaigns to urge the manufacturers to reduce prices and thereby allow health systems to provide these medicines to patients to prevent and mitigate further disability.^4^ In a different example of a public health protection measure, COVID-19 vaccines have the potential to protect people with disabilities, often who are already in vulnerable conditions and situations, from a COVID-19 infection aggravating their existing impairments, including by protecting against Long COVID and the incapacity it incurs.^5^ When they first entered the market, COVID-19 vaccine doses were priced higher in markets less able to pay (e.g., South Africa) compared to wealthier markets (e.g., the EU).^6^ These examples illustrate how many medicines remain inaccessible (due to high prices and/or supply restrictions) for people who need them. This problem becomes all the more egregious considering that it disproportionately affects people in low- and middle-income countries where people with disabilities experience more negative consequences as a result of these systemic challenges (i.e., high medicine prices and low availability) “due to their impairments and disabled status, and likely overall greater healthcare needs and higher levels of poverty and exclusion.”^7^ These examples offer evidence of the global consequences of failing to regulate transnational pharmaceutical company practices for the protection and promotion of human health — consequences which are borne unduly by people with disabilities who are already in vulnerable positions in society.

Regional international organizations, such as the EU, play an ever more important role in the governance of human health and access to medicines despite the relatively little attention paid to them in academic scholarship.^8^ Regional international organizations promote cooperation amongst the countries in a particular world region in selected policy areas, often relevant to human health. The EU is an example of far-reaching cooperation under the auspices of a regional international organization, resulting in the integration of markets and harmonization of rules across countries for the free movement of goods, persons, capital, and services on that market. While the EU’s internal market governance and activities undoubtedly influence human health *within* the Union, attention is now being paid to the potential influence of the EU’s internal governance on human health *externally.* ^9^ A range of policies and actions of the EU’s institutions and agencies have extraterritorial effects on people’s access to medicines, health, and well-being, particularly in low- and middle-income countries (LMICs).^10^

When it comes to the protection of human health in the internal market, EU lawmakers have placed legal limits on the scope of product marketing and company conduct, inevitably affecting the single market, to protect public health throughout the Union.^11^ However, in relation to its extraterritorial influences on human health, the EU generally lacks the jurisdiction over foreign markets that is required for the Union to adopt laws for the targeted legal protection of human health externally.^12^

Although the application of EU law is usually limited to the territory of the EU, the EU’s regulatory norms, rules, and arrangements related to pharmaceuticals laid down in Union law continue to travel across international borders with few legal protections for human health following them. The EU’s international regulatory influence over pharmaceuticals touch, for example, third countries’ decisions about or regulation of intellectual property protection and market exclusivities granted to certain medicines, market licensing/approval of certain medicines, and the definition of and regulatory or other support granted to certain categories of medicines (e.g., orphan medicines).^13^ To illustrate the scope of the EU’s impact, consider that 380 million people in Latin America are served by local regulatory agencies that rely on the European Medicines Agency (EMA)’s evaluations and/or the EU’s market authorization decisions (among other reference regulators) when deciding to license new chemical entities on their local markets.^14^ Yet, despite the demonstrable impact of the global diffusion of the EU’s norms, rules, and arrangements for pharmaceutical regulation on human health, the Union is acting without a clear distillation of its legal responsibilities for protecting human health externally.

The issue of the EU’s extraterritorial obligations toward human rights is a perennial question that has scarcely — if ever — been squarely addressed in the health domain.^15^ The EU institutions bear responsibility to consider a high level of protection for human health across all their policies and activities (without explicit territorial limitation).^16^ These responsibilities are guided by the EU’s values and objectives in its relations with the wider world, such as the principles of equality and solidarity, the universality and indivisibility of human rights, respect for human dignity, and respect for the principles of the UN Charter and international law.^17^ However, these values and objectives, in themselves, offer little concrete guidance to operationalize the EU’s responsibilities with regard to health or specifically global medicines access.^18^

Elaborating a framework for the legal responsibilities of the EU towards extraterritorial medicines access is complicated by the puzzle of overlapping yet distinct layers of international and European Union law involved. The EU’s legal responsibilities will inevitably be defined by laws that apply legal obligations to the EU regarding the health of persons outside the Union. Identifying relevant laws that satisfy these criteria is a challenge. For example, the European Convention on Human Rights (ECHR) currently occupies an unclear position in the EU’s legal order,^19^ and the instrument’s definition of jurisdiction does not catch most situations where the EU’s actions are producing effects on access to medicines extraterritorially. In the EU legal order, the Charter of Fundamental Rights of the EU applies to the acts and policies of the Union’s institutions. Although the Charter enshrines rights that have been applied extraterritorially in the past,^20^ the instrument articulates a right to health that gives a wide margin of appreciation to national law and policy, complicating the adjudication of the EU’s role in health protection.^21^ In the scope of international law, the EU, as a regional international organization, is not a contracting party to most UN human rights treaties (except the Convention on the Rights of Persons with Disabilities (CRPD)) that enshrine extraterritorial duties towards health rights. Even though there is value in exploring the EU’s extraterritorial obligations arising indirectly from international law, such duties occupy a lesser position in the EU’s legal order than directly binding obligations from instruments to which the EU is a contracting party.^22^

The case of access to medicines for people with disabilities in developing countries poignantly illustrates two questions: (1) what are the EU’s normative legal responsibilities towards *external* human health protection; and (2) to what extent have the Union’s policies and activities lived up to its legal responsibilities? This paper asserts the potential of the CRPD — the only UN human rights treaty to which the EU is a contracting party — to create a legal obligation recognized in the EU legal order for the Union to take steps toward extraterritorial medicine access for people with disabilities. These questions arise on the coattails of the 2020 Pharmaceutical Strategy for Europe^23^ and the extensive revision of the Union’s general pharmaceutical legislation, both of which will shape the EU’s market and the Union’s global influence over foreign pharmaceutical markets — and by extension, foreign health systems — for decades to come.^24^ Moreover, attention is increasingly turning to the role of regional international organizations in advancing international (human rights) law and their effects on human health.^25^

This paper first introduces the CRPD, the intersection of health and disability as laid down in the instrument, and the content and contours of the legal obligations flowing from it with regards to extraterritorial access to medicines as part of health rights. Then, this paper elucidates the EU’s obligations under the CRPD in light of the Union’s competences in fields related to medicines and evaluates to what extent the EU has met its obligations in two cases: one, the EU’s joint procurement agreements for COVID-19 vaccines (an internal market measure), and two, Team Europe’s Manufacturing and Access to Vaccines, Medicines and Health Technologies (MAV+) program benefitting Africa (an external measure).

## 2. Convention on the Rights of Persons with Disabilities

In 2008, the CRPD entered into force, ushering in renewed potential for the protection and promotion of the human rights of persons with disabilities around the globe. Although foregoing UN human rights treaties extended human rights protections to people with disabilities, those rights and protections were often denied, thereby necessitating a unique instrument (i.e., the CRPD) drawing attention to and promoting targeted interventions for states to empower people with disabilities to enjoy their rights.^26^ Embodying the notion of “nothing about us without us,” the negotiation process leading to the CRPD’s adoption involved persons with disabilities as members of nongovernmental organizations (present for the first time during negotiations), as well as members of state delegations and UN organizations.^27^ This inclusivity is credited with generating sizable global support from 143 signatory states and 71 ratifying states (in 2009).^28^ As such, the CRPD was touted as the “first legally binding international instrument that specifically protects the rights of some 650 million people worldwide.”^29^

Currently, the CRPD has 191 contracting parties, including, notably, states and regional integration organizations. This is significant for two reasons. First, the inclusion of regional integration organizations as potential contracting parties signals a broader view on the global governance of human rights than previous UN human rights treaties that state parties have agreed to. A regional integration organization (RIO) is defined by the CRPD as an organization composed of sovereign states from a particular world region where those member states have transferred competences to the RIO on matters governed by the CRPD.^30^ Second, having 191 contracting parties suggests a significant global consensus on the need for this legal instrument and the importance of commitments therein to the realization of disability rights.^31^

Contracting parties have the general legal obligation under the CRPD to adopt “all appropriate legislative, administrative and other measures” for implementing Convention rights, to consider “the protection and promotion of human rights for persons with disabilities in all policies and programmes,” to “ensure that public authorities and institutions act in conformity with the present Convention.”^32^ Importantly for this consideration of medicines in the context of disability rights, contracting parties have general legal obligations to:

… undertake or promote research and development of, and to promote the availability and use of new technologies, including … devices and assistive technologies, suitable for persons with disabilities, giving priority to technologies at an affordable cost.^33^

As explained below (in subsection 3.3.1), the terms and conditions for product access and availability can be established already at the research and development stage. Entities (whether public or private) that are involved in facilitating, financing, and/or coordinating research and development into new technologies, including medicines, can and should leverage their position in the innovation process and attach conditions for the affordability and access to the resulting technologies that are suitable for persons with disabilities. Any contracting party to the CRPD that does so is taking steps aligned with its general legal obligation above to promote the research and development of technologies for people with disabilities.

In the context of economic, social, and cultural rights, contracting parties are obliged to take steps within their maximum available resources and “where needed, within the framework of international cooperation” to progressively realize Convention rights.^34^ From this provision, contracting parties have the primary responsibility for realizing Convention rights to the extent that their resources allow, and through international cooperation, where required. By citing international cooperation among the general legal obligations, the Convention already alerts contracting parties to the fact that international cooperation has a role to play in the Convention’s operationalization (as will be seen in CRPD article 32).

Before turning to an examination of the international cooperation component of the CRPD (section 2.3), the following subsection 2.1 examines the relationship between human health and disability, as the latter is conceptualized in the CRPD. This examination helps reveal to what extent the rights and obligations enshrined in the CRPD relate to access to medicines as part of the right to health (subsection 2.2).

### 2.1. Relationship Between Health and Disability

The Preamble of the CRPD defines disability as:

… an evolving concept and that disability results from the interaction between persons with impairments and attitudinal and environmental barriers that hinders their full and effective participation in society on an equal basis with others^35^

Critical in this definition is its broad nature and composition of two components: people with impairments on one hand, and the attitudinal and environmental barriers on the other hand. CRPD article 1 sketches the boundaries of impairments as “long-term physical, mental, intellectual or sensory impairments.”^36^ This definition is comparable to the World Health Organization (WHO)’s International Classification of Functioning, Disability and Health (ICF) understanding of disability as denoting:

… the negative aspects of the interaction between an individual (with a health condition) and that individual’s contextual factors (environmental and personal factors).^37^

The ICF seeks to provide a “unified and standard language and framework for the description of health and health-related states.”^38^ Drawing out this similarity between the definition of disability in the CRPD and the WHO’s ICF illustrates how intrinsically linked the experience of a disability is to one’s health status.

### 2.2. Access to Medicines as Part of the Right to Health of People with Disabilities

Human rights are interdependent and indivisible. Although numerous rights in the CRPD relate to and support the highest attainable standard of physical and mental health for disabled persons, CRPD article 25 articulates most clearly the right to health. It requires contracting parties to recognize that persons with disabilities have the right to the enjoyment of the highest attainable standard of health without discrimination based on disability. Moreover, contracting parties shall take all appropriate measures to ensure access for persons with disabilities to health services. Importantly for the present article, there are two measures contracting parties should address:

a) Provide persons with disabilities with the same range, quality and standard of free or affordable health care and programs as provided to other persons, including in the area of sexual and reproductive health and population-based public health programs;

b) Provide those health services needed by persons with disabilities specifically because of their disabilities, including early identification and intervention as appropriate, and services designed to minimize and prevent further disabilities, including among children and older persons;^39^

The first measure concerns providing a person with a disability with the healthcare, such as essential medicines, required for the enjoyment of their right to health. Medicines should be provided without discrimination against people with disabilities, and in “the same range, quality, and standard of free or affordable healthcare” as other people enjoy. The medicines to be provided need not be directly related to the person’s disability/disabilities. The type of medicines to be provided may relate to any or all aspects of realizing the right to health, as indicated by the Convention’s framers, who specified the examples of healthcare for sexual and reproductive health and population health programs. In other words, this measure requires contracting parties to provide the full range of essential medicines (as defined by WHO and interpreted to be a part of the right to health in other UN human rights treaties^40^) to people with disabilities, including medicines that are unrelated to a manifest impairment but rather are linked to certain physiological functions of the human body and/or preventative healthcare, such as contraception, oxytocin at childbirth, and vaccines.

The second measure concerns the provision of healthcare, including essential medicines, that are required by people with disabilities “specifically because of their disabilities.” This type of healthcare may be related to early intervention at the onset of a disability, and serves to “minimize and prevent further disabilities” once a disability has already manifested. Over the years, essential medicines for certain conditions satisfy these criteria. One example is of sofosbuvir, a breakthrough antiviral treatment for progressively debilitating hepatitis C infections.^41^ Before the launch of sofosbuvir and other direct-acting antiretrovirals, hepatitis C patients were treated with interferon-based therapies by injection, causing significant and sometimes draining side effects.^42^ Another example is of genetic and/or rare conditions such as hemophilia, where patients may bleed excessively after (minor) injury, which is a risk that significantly impacts their quality of life. Supplementing the patients’ deficiencies in clotting factors VIII and IX, which are essential medicines recognized by WHO and some countries, is important prophylaxis for patients to manage the condition and prevent complications.^43^ Other examples may include lifesaving essential medicines, such as insulin for type 1 diabetes (caused by a physical impairment of the pancreas) and naloxone for opioid addiction (which is considered a disability because it limits a person’s daily activity). In this way, a disability rights lens applied to the persistent inequitable access to medicines globally can help refocus attention on unmet medical needs worldwide.

In conclusion, contracting parties to the CRPD are obliged to provide access to health services, including essential medicines, required for people with disabilities for the full enjoyment of their right to health. Additionally, contracting parties must provide access to essential medicines required by a person’s specific disability/disabilities to minimize or prevent further disability.

### 2.3. Disability Rights and Obligations Across Borders

The CRPD contains a general obligation on all contracting parties for the progressive realization of economic, social, and cultural rights to the maximum of the parties’ available resources and “where needed, within the framework of international cooperation.”^44^ Coupling the general obligation of progressive rights realization with international cooperation strongly suggests that the primary limit on the extent of a contracting party’s duties is the party’s own resource availability and not the spatial or geographic territory in which rightsholders are located, which is a similar approach as is taken in the International Covenant on Economic, Social and Cultural Rights (ICESCR).^45^

In contrast to the territorial limits enshrined in the International Covenant on Civil and Political Rights,^46^ the CRPD contains an explicit article on international cooperation which obliges states to “recognize” its importance and “undertake appropriate and effective measures” between and among states, and in partnership with international and regional organizations and civil society for undertaking certain measures.^47^ Article 32 of the CRPD provides a non-exhaustive list of steps that contracting parties could take to discharge their obligations enshrined in that article. Those steps of relevance for the present article include:

b. Facilitating and supporting capacity-building, including through the exchange and sharing of information, experiences, training programs and best practices;

c. Facilitating cooperation in research and access to scientific and technical knowledge;

d. Providing, as appropriate, technical and economic assistance, including by facilitating access to and sharing of accessible and assistive technologies, and through the transfer of technologies.^48^

These are the steps that contracting parties should take for the realization of Convention rights. When read together with other relevant articles, such as the right to health, these obligations can be understood in the context of this paper as requiring contracting parties to facilitate and support capacity building and research cooperation for the provision of quality and affordable health services and medicines to people with disabilities.^49^ One example of this duty can include capacity-building foreign regulators to safeguard the quality, safety, and efficacy of a medicine’s supply abroad. This duty is particularly incumbent when a specific medicine, needed by people with disabilities, is prone to problems with quality, contamination, or adulteration.^50^ Moreover, contracting parties would be required to provide technical and economic assistance and technology transfer for the provision of the same range, quality, and affordability of health services and medicines available to people without disabilities.^51^ Engaging in technology transfer to provide medicines as assistive devices to people who require specific pharmaceuticals to minimize or prevent further disability is also an example of measures contracting parties should take.^52^

The following section explores how these obligations apply to the EU.

## 3. EU’s Obligations Under CRPD

In December 2010, the EU made history when it acceded to the CRPD alongside all EU Member States. This event marked the first UN human rights treaty to which any regional integration organization, and specifically the EU, had become a contracting party.^53^ The CPRD Committee commended the EU’s commitment as “a positive precedent in public international law.”^54^

EU accession to the CRPD was based on the Union’s competence to facilitate the functioning of the internal market and to combat discrimination, including on grounds of disability.^55^ Together, these legal bases suggest EU lawmakers saw accession to the CRPD as a vehicle to address discrimination based on disability and to promote the rights of people with disabilities in the EU’s single market. International agreements that the EU has concluded, such as the CRPD, form an integral part of the EU legal order.^56^ As such, the CRPD is binding on EU institutions, albeit falling lower in the hierarchy of norms than primary EU law. Within the EU’s legal order, the CRPD applies to the policy areas of the Union insofar as the Member States have delegated their powers in those areas to the EU. The following subsection 3.1 explores the EU’s competences (or delegated powers) with regard to health and medicines.

### 3.1. EU Competences Regarding Health and Medicines

The protection of human health touches multiple policy fields. In some of these fields the EU enjoys full competence, while in others the EU’s powers are limited to complementary or parallel competences with its Member States (explained below). The obligations imposed by the CRPD on the EU track the Union’s competences in the respective areas.^57^ In other words, the EU cannot discharge the obligations in the CRPD if the Union has not been delegated the legal powers to act in that policy area.

The EU has a complementary competence for the protection of human health, which means that EU Member States retain the right to organize and finance their own health systems as they see fit while the EU may only “support, coordinate or supplement” the actions of Member States.^58^ Nevertheless, a high level of human health protection is an overarching aim of the Union to be considered across all EU policies and actions without an explicit territorial restriction.^59^ Although the EU holds complementary competences for a limited scope of activities in the field of public health, it is exceptionally permitted to adopt internal market regulations and measures to harmonize national laws for the safety of the Union’s pharmaceutical supply.^60^ Such actions are also closely linked with the EU’s full competence to promote the functioning of the internal market, including through regulating the safety and quality of the pharmaceutical supply.^61^

The EU shares competences with its Member States in two sectors relevant for access to health technologies: international cooperation and humanitarian aid policy on one hand, and research and innovation policy on the other. With regard to international cooperation and humanitarian aid, the EU holds parallel competences in this domain alongside its Member States, meaning that the steps taken by both parties should “complement and reinforce each other.”^62^ Importantly, the EU’s development cooperation policy has the primary objective of reducing and eradicating poverty, which empirical evidence has linked with measures for the affordability and availability of essential medicines.^63^ In the field of research and innovation policy, the objective of the EU’s actions is to, as a region, “become more competitive, including in its industry” while also promoting research activities considered necessary to support the other Union objectives elaborated in Treaty on the Functioning of the European Union (TFEU).^64^ Promoting research on health technologies needed by people in LMICs to reduce and alleviate poverty, thereby advancing the EU’s development policy objective enshrined in the TFEU, would also contribute towards the objectives of the EU’s research and innovation actions.

Therefore, by acceding to the CRPD, the EU is obliged to facilitate and support capacity building and research cooperation and provide technical and economic assistance through international cooperation (within the extent of the Union’s competences in these areas) for the realization of Convention rights.

### 3.2. Monitoring the EU’s Duties to Protect the Health of People with Disabilities

As a contracting party to the CRPD, the EU is subject to the Convention’s treaty monitoring system, which is similar to systems in other UN human rights treaties.^65^ The monitoring procedure is overseen by the Committee on the Rights of Persons with Disabilities (hereafter “the Committee”), which is responsible for assessing reports from contracting parties about the steps they have taken to implement their obligations under the Convention.^66^ Contracting parties are expected to report regularly (i.e., at least every four years) on their progress in the form of a comprehensive initial report, which is considered by the Committee also in light of shadow reports provided by non-state actors.^67^ Following the opportunity for the contracting party to provide clarifications requested by the Committee, it issues observations and recommendations on the contracting party’s report.^68^

The EU’s external actions towards access to quality medicines have been raised in a foregoing report by the Union to the Committee. In the Committee’s 2015 Concluding Observations on the EU’s actions, the Committee recommends that the Union “take measures to ensure access to quality health care for all persons with all types of disabilities.”^69^ In the absence of an explicit definition by the CRPD Committee, “quality” health care can be understood through the authoritative interpretation of the right to health by the UN Committee on Economic, Social and Cultural Rights (UN CESCR). The UN CESCR is the treaty monitoring body of the ICESCR, which has issued authoritative interpretations of the right to health, encompassing the provision of access to essential medicines defined by WHO.^70^ The UN CESCR has stated that quality health care is “culturally acceptable, … scientifically and medically appropriate and of good quality” which in general requires access to “scientifically approved and unexpired drugs,” among other health facilities, goods and services.^71^

In a subsequent report to the Committee on its own implementation of the CRPD, the EU stated in 2021 that its own activities (i.e., in the context of the EU4Health Work Programme) aim to advance the health rights of people with disabilities by supporting Member States to improve “access to healthcare for persons with disabilities” on the internal market.^72^ From an external perspective:

“[t]he EU works on improving access to quality care for persons with disabilities as part of universal health coverage in countries where it cooperates on health or supports social protection.”^73^

These commitments by the Union, taken together with the authoritative definition of quality healthcare under the UN human rights treaties, can be understood as a policy commitment of the EU to take steps to improve access to scientifically approved (or licensed), medically appropriate, unexpired medicines of good quality as part of its universal health coverage efforts in third countries.

In the same report, the EU responded to issues regarding its attention to people with disabilities within its COVID-19 response. Notably, the European Commission reported having “secured access to new vaccines and supported Member States in their rollout, calling on them to define priority groups for vaccination [within the Union].”^74^ While these activities are beneficial for people with disabilities within the Union, the Commission’s report neglected to explain whether or how the EU considered vaccine access and rollout for people living with disabilities beyond the EU’s borders. Moreover, the Commission’s report specifically omitted any discussion of how the EU’s own large-scale procurement of COVID-19 vaccines would (negatively) impact the limited global vaccine supply at that time, resulting in fewer available doses for people in LMICs, and what impact that would have on vaccine prioritization and access in LMICs.

### 3.3. Analysis of EU Obligations to Advance Disability Rights and Access to Medicines

Among the range of EU policy areas touching on access to medicines, this paper selects two areas to further examine to what extent the Union has discharged its duties to facilitate access to knowledge, sharing technologies, and providing assistance internationally in support of access to medicines for people with disabilities. One of the cases is the EU’s joint procurement agreements for COVID-19 vaccines for distribution on the internal market (subsection 3.3.1). The second case is the EU’s Manufacturing and Access to Vaccines, Medicines and Health Technologies (MAV+) program, which is part of the Union’s external work in the Neighborhood, Development, and International Cooperation fields (subsection 3.3.2). In both cases, the EU’s actions have a significant influence on the upstream availability and affordability of the vaccine supply beyond the Union’s borders. Although the prioritization of vaccine distribution to specific population groups is a matter of national policy in LMICs, the pressure on national decision-makers to limit vaccine access, and consequently potentially discriminating against people with disabilities, is heightened in the face of low global supplies. Experience during the COVID-19 pandemic showed that despite WHO’s guidelines for health authorities to consider people with disabilities when establishing vaccine prioritization plans,^75^ national or regional vaccine allocation plans insufficiently prioritized people with disabilities due, in part, to shortages of vaccines.^76^ Consequently, some people with disabilities who received lower priority on vaccination waiting lists were further marginalized and faced a heightened risk of contracting a COVID-19 infection (thereby placing them also at higher risk of developing a long COVID-19 infection and the ongoing incapacity that it incurs). Therefore, actions by the EU that are consistent with its obligations under the CRPD (explained in section 2) could help alleviate global medicines supply shortages and avoid discriminatory rationing at a local level, which can negatively impact the health and well-being of people with disabilities. The following subsections examine two cases and the extent to which the EU has acted in line with its obligations under the CRPD.

#### 3.3.1. EU Joint Procurement Agreements for COVID-19 Vaccines

The EU’s joint procurement of COVID-19 vaccines on behalf of Member States shows how the Union’s internal market activity can have potential impacts on the availability and affordability of vaccines on the global market, and thereby influence the realization of disability rights abroad.^77^ The legal basis for the EU’s joint procurements of medical supplies such as vaccines is the EU Health Threats Decision on cross-border health threats and the Joint Procurement Agreement (JPA) with EU Member States.^78^

The European Commission, representing EU Member States that voluntarily participated in the joint procurement, coordinated and concluded several agreements with pharmaceutical manufacturers for the supply of COVID-19 vaccine doses to members of the JPA. This type of agreement was an “advanced purchase agreement (APA)” that serves to stimulate and de-risk the late-stage development of products needed by society but that have historically lacked the market potential or profit incentive for developers.^79^ The contractor, in this case the European Commission, contributes funds to end-stage product development, and is in a sense a co-investor. As such, through the APA the EU has the potential to also establish the terms and conditions for the management of the knowledge, data, and intellectual property (IP) generated through the EU’s investment (including sharing and transfer of that knowledge, data, and IP), and the availability and affordability of the end medical product.^80^ This potential was further supported by text in the 2020 agreement between the European Commission and its Member States for the procurement of COVID-19 vaccines. Annex A to that Decision approving the purchase agreement included the commitment:

In the negotiations with the pharmaceutical industry under the present Agreement, the Commission will promote a COVID-19 vaccine as a global public good. This promotion will include access for low and middle income countries to these vaccines in sufficient quantity and at low prices.

The Commission will seek to promote related questions with the pharmaceutical industry regarding intellectual property sharing, especially when such IP has been developed with public support, in order to these objectives.^81^

Observed in the first paragraph, the term “global public good” refers to goods (both tangible and intangible, such as clean air) that are available to all people and where the enjoyment of these goods by one person does not exclude or diminish the benefits that other people can enjoy.^82^ To discharge their “global public goods” commitment, the European Commission and EU Member States would have to take steps to regulate the management and sharing of the IP, knowledge, and data generated about COVID-19 vaccines as part of these purchase agreements. The commitments in Annex A to promote access to COVID-19 vaccines “in sufficient quantity and at low prices” in developing countries are congruent with the EU’s obligation to take steps through international cooperation that would ultimately provide people with disabilities the same range, quality, and standard of affordable public health care (i.e., vaccine access) as provided to other people.^83^ Moreover, by promoting IP sharing in relation to COVID-19 vaccines, the Commission advances its obligation to facilitate access to scientific and technical knowledge and the sharing of the IP underlying accessible technologies.^84^

However, the European Commission’s discharge of these agreements reveals a departure from the above obligations under the CRPD. Pascale Boulet and colleagues’ analysis of the three purchase contracts between the European Commission on one hand, and AstraZeneca, Moderna, and CureVac on the other, show that where the text is not redacted, the contracts permit the respective pharmaceutical manufacturers to retain ownership over the IP, including the know-how required to scale up vaccine production by other manufacturers and the data generated in the context of the agreement.^85^

This case illustrates that failing to condition the EU’s purchases of vaccines on the sharing (be it through licensing or otherwise) of the IP, knowledge and data necessary for other manufacturers to produce COVID-19 vaccines in other parts of the world is a breach of the duty to facilitate cooperation in research and access to scientific and technical knowledge (CRPD article 35(c)) for the provision of health services to people with disabilities (CRPD article 25(a)).

#### 3.3.2. Team Europe’s BioNTainers for Manufacturing mRNA Vaccines in Africa

Initiated in 2021, the objective of the EU’s MAV+ program is to facilitate access to vaccines, medicines, and technologies in Africa by supporting the scale-up of local vaccine manufacturing capacity, among other strategies.^86^ The EU’s specific role, alongside 16 of its Member States and the European Investment Bank, is to provide financial and technical support for the supply and demand aspects, and for shaping an environment conducive to local manufacturing.^87^ An important part of the MAV+ program is currently the EU’s collaboration with the German pharmaceutical company BioNTech where the latter entirely finances and delivers mobile units, called BioNTainers, to sites in Africa for manufacturing mRNA vaccines.^88^ BioNTainers are touted as providing a “modular system, scalable and turnkey solution for local manufacturing” capacity.^89^ The first BioNTainer was delivered and launched in Rwanda in 2023 with funding from the company BioNTech and financial and technical support from the EU to the Rwandan regulators to shape a local business environment supportive of vaccine manufacturing. Parwani notes in her analysis that through the MAV+ program the EU provides public funds for capacity-building the local regulator, for local workforce training, and for “fostering a start-up ecosystem in the country.”^90^ These activities are consistent with the EU’s obligations under the CRPD to facilitate and support capacity building and research cooperation for the provision of quality healthcare (i.e., quality-assured and appropriately regulated vaccines).^91^

While some of these activities promoting access to vaccines in third countries appear to be highly congruous with the EU’s obligations towards international cooperation for disability rights under the CRPD, further examination reveals some discordance. Parwani’s critique reveals that the BioNTainer’s “cut-and-paste model” simply exports manufacturing units to regions with limited production capacity.^92^ She notes that currently there are no binding obligations for BioNTech to share its technology with other manufacturers.^93^ BioNTech’s model thereby sidesteps the important, and likely more sustainable, act of collaborating with existing African manufacturers to build their capacities and working with licensing agreements to ensure technology transfer to the emerging local ecosystem of manufacturers.^94^ Therefore, through the BioNTainer initiative, the EU’s MAV+ program could take further steps to support technology transfer for subsequent (local) access to and sharing of technologies for the production of an adequate and affordable supply of mRNA vaccines serving local populations with disabilities in Rwanda or on the African continent, as required under CRPD articles 25(a) and 32(d).

## 4. Conclusion

Access to essential medicines is critical for realizing the health rights and other human rights of people with disabilities. In a globalized medicines market, regional international organizations, such as the EU, play an increasingly prominent role in promoting, facilitating, and in some cases, providing access to essential medicines for people with disabilities. As the EU has acceded to the CRPD, the Union bears legal responsibilities for realizing the health rights of people with disabilities, including providing healthcare such as medicines, through various measures including international cooperation. The cases of the EU’s joint procurement of COVID-19 vaccines for the internal market and its external BioNTainer initiative to facilitate vaccine products in Africa allow for a deeper assessment of the extent to which the EU discharges its duties towards the extraterritorial health rights of people with disabilities. This analysis has shown that, although the EU has made commitments in these cases consistent with its obligations under the CRPD, the practical discharge of these commitments falls short of Convention standards. Specifically, the EU should take further steps to align its policies and actions for intellectual property sharing and technology transfer of vaccine-related knowledge and know-how with other manufacturers, which is needed to build and maintain vaccine manufacturing capacity in regions around the world. By taking steps to bolster foreign vaccine production and global vaccine supplies, the EU can reduce the risk of discriminatory vaccine rationing and prioritization in LMICs that negatively impacts people with disabilities.
